# Tubulin Polymerization Promoting Proteins (TPPPs) of Aphelidiomycota: Correlation between the Incidence of p25alpha Domain and the Eukaryotic Flagellum

**DOI:** 10.3390/jof9030376

**Published:** 2023-03-19

**Authors:** Ferenc Orosz

**Affiliations:** Research Centre for Natural Sciences, Institute of Enzymology, 1117 Budapest, Hungary; orosz.ferenc@ttk.hu

**Keywords:** *Amoeboaphelidium protococcorum*, Aphelidiomycota, flagellum, fungi, Olpidiomycota, *Paraphelidium tribonematis*

## Abstract

The seven most early diverging lineages of the 18 phyla of fungi are the non-terrestrial fungi, which reproduce through motile flagellated zoospores. There are genes/proteins that are present only in organisms with flagellum or cilium. It was suggested that TPPP-like proteins (proteins containing at least one complete or partial p25alpha domain) are among them, and a correlation between the incidence of the p25alpha domain and the eukaryotic flagellum was hypothesized. Of the seven phyla of flagellated fungi, six have been known to contain TPPP-like proteins. Aphelidiomycota, one of the early-branching phyla, has some species (e.g., *Paraphelidium tribonematis*) that retain the flagellum, whereas the *Amoeboaphelidium* genus has lost the flagellum. The first two Aphelidiomycota genomes (*Amoeboaphelidium protococcorum* and *Amoeboaphelidium occidentale*) were sequenced and published last year. A BLASTP search revealed that *A. occidentale* does not have a TPPP, but *A. protococcorum*, which possesses pseudocilium, does have a TPPP. This TPPP is the ‘long-type’ which occurs mostly in animals as well as other Opisthokonta. *P. tribonematis* has a ‘fungal-type’ TPPP, which is found only in some flagellated fungi. These data on Aphelidiomycota TPPP proteins strengthen the correlation between the incidence of p25alpha domain-containing proteins and that of the eukaryotic flagellum/cilium.

## 1. Introduction

Avidor-Reiss et al. [1] previously suggested, based on bioinformatics analysis, that there are some genes/proteins that are present only and exclusively in organisms with flagella or cilia. Cilia (flagella) are microtubule-based cellular extensions of a sensory and/or motile function. The collection of these genes composes the ciliome. Genes of the ciliome are generally absent in species without cilium/flagellum. The flagellum and the cilium are basically the same microtubule-based organelle and are usually distinguished by their number and length [2].

**Figure 1:**
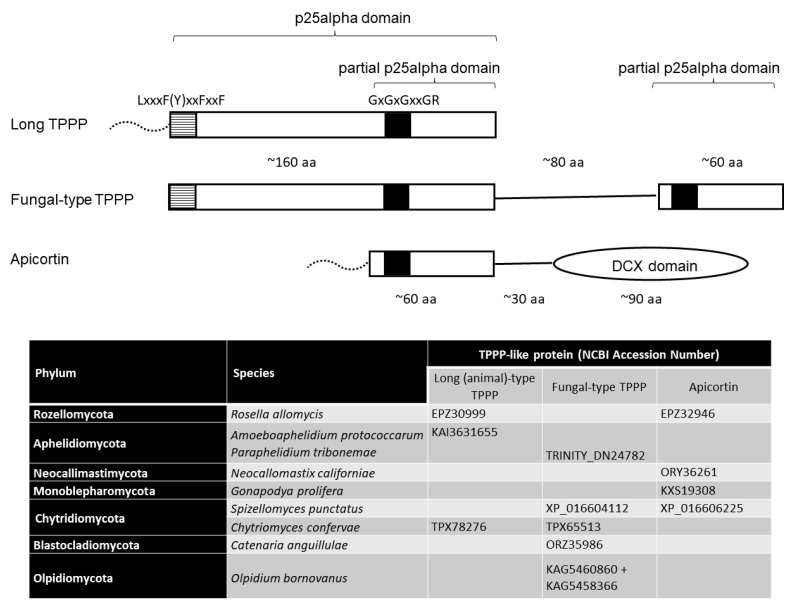
Schematic structure of TPPP-like proteins and their occurrence (selected examples) in non-terrestrial fungi ([6,9] and this paper). Black and dashed line squares indicate highly conservative sequence motifs. Dotted lines represent disordered regions of various length which are present in some species. aa- amino acids.

TPPP (Tubulin Polymerization Promoting Protein) is a microtubule-stabilizing protein containing a p25alpha domain (Pfam05517 or IPR008907) [3]. It is not a structural domain but was generated automatically from a sequence alignment from Prodom 2004.1 for the Pfam-B database. I proposed that the TPPP protein also belongs to the ciliome based on the sequence data available at that time [4]. Later, I modified this suggestion so that the assumption would also be valid for ‘TPPP-like proteins’, which contain at least one complete or partial p25alpha domain [5]. The members of the family of TPPP-like proteins differ from each other in the completeness of the p25alpha domain (long, short, truncated, partial) and in the presence or absence of other domains (e.g., DCX or EF-hand) [5]. A distinct ‘fungal-type’ TPPP, which is found in some flagellated fungi, contains both a complete and a partial p25alpha domain [6] (Figure 1). An essential role of TPPP in the formation of flagella was demonstrated in *Chlamydomonas reinhardtii*, biflagellated green algae, through the use of null mutant of FAP265, its TPPP ortholog [7]. Very recently, it has also been shown that TPPP (Py05543) is required for male gametocyte exflagellation in *Plasmodium yoelli* [8].

Fungi consist of 18 phyla according to the latest classification by Tedersoo et al. [10]. Among these, the seven early-branching clades are the non-terrestrial fungi, which reproduce by using motile flagellated zoospores. In terrestrial fungi, the flagellum is lost. Thus, fungi provide an ideal opportunity to test and confirm the hypothesis of the correlation between the occurrence of the p25alpha domain and that of the eukaryotic cilium/flagellum since the flagellum occurs in some phyla and not in others.

Earlier, I found that of the seven phyla of flagellated fungi, five had one or more TPPP-like proteins [6]. These phyla are Rozellomycota, Neocallimastigomycota, Monoblepharomycota, Chytridiomycota, and Blastocladiomycota (Figure 1). Among the two phyla without TPPP-like proteins, Aphelidiomycota and Olpidiomycota, as complete genomes, were not available; thus, I predicted that if this situation changed, it could be shown that they also possess p25alpha domain-containing proteins. Recently, Chang et al. [11] published the genome of an Olpidiomycota, *Olpidium bornovanus*, which contained a fungal-type TPPP designated as hypothetical partial proteins, KAG5460860 and KAG545836. I have shown that they are parts of a single protein [9]. Thus, only the Aphelidiomycota phylum lacked data regarding proteins with a p25alpha domain. 

## 2. Materials and Methods

A database homology search was carried out with an NCBI Blast search [12] (http://www.ncbi.nlm.nih.gov/BLAST/): sequences of various fungal proteins (e.g., *Batrachochytrium dendrobatidis* XP_006680205, *Chytriomyces confervae* TPX65513, *Powellomyces hirtus* TPX57673, *Spizellomyces punctatus* XP_016604112) containing p25alpha-domain were used as queries against protein databases to find similar sequences in the *Amoeboaphelidium* genus using BLASTP analysis. Version 1.5 of the predicted *Paraphelidium tribonematis* proteome was downloaded from https://figshare.com/articles/dataset/Commun_Biol_aphelid_datasets/7339469/1 (accessed on 14 November 2022) [13] and searched for TPPP-like proteins. The recent phylogenetic classification by Tedersoo et al. [10] was followed. The species names ‘*Amoeboaphelidium protococcorum*’ and ‘*Paraphelidium tribonematis*’ were used instead of ‘*Amoeboaphelidium protococcarum*’ and ‘*Paraphelidium tribonemae*’ based on the Latin rule [14]. The accession numbers of protein sequences refer to the NCBI GenBank database.

Multiple alignments of sequences were conducted by the Clustal Omega program [15]. Bayesian analysis, using MrBayes v3.1.2 [16], was also performed to construct a phylogenetic tree using whole sequences of TPPP proteins. Default priors and the WAG model [17] were used, assuming equal rates across sites. Two independent analyses were run with three heated and one cold chain (temperature parameter 0.2) for generations, as indicated in Figure legends, with a sampling frequency of 0.01, and the first 25% of generations were discarded as burn-in. The two runs were convergent.

## 3. Results and Discussion

Mikhailov et al. [18] recently published the genome of *A. protococcorum* and *Amoeboaphelidium occidentale*. The ancestor of Aphelidiomycota was flagellated, and in some cases, a reduction in flagellum occurred as in *A. occidentale* and *A. protococcorum*, while, in other species, it was retained (e.g., *P. tribonematis*) [18]. Mikhailov et al. [18], based on the data from [19], showed that the *P. tribonematis* transcriptome demonstrated the conservation of ciliogenesis and axonemal motor proteins, in accordance with the presence of flagellated zoospores, while the *Amoeboaphelidium* species lost most of their genes/proteins. Interestingly, despite the loss of the flagellum, *A. occidentale* possessed most of the components of the intraflagellar transport (IFT), which are perhaps the most characteristic flagellar proteins; however, *A. protococcorum* has only a few of them.

**Figure 2:**
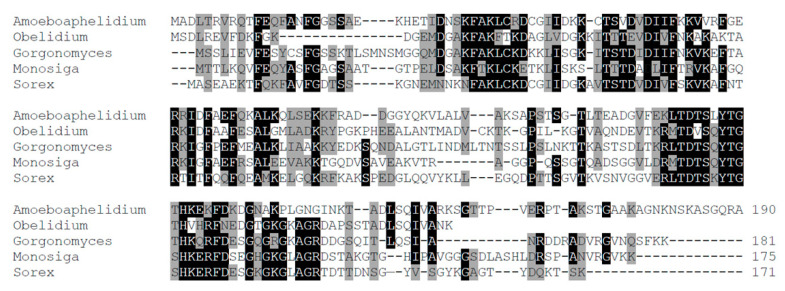
Comparison of the sequence of *Amoeboaphelidium protococcorum* TPPP with those of some long (animal)-type TPPPs. Multiple sequence alignment (manually refined) was conducted by Clustal Omega [15]. Amoeboaphelidium, *A. protococcorum* strain X5 KAI3631655 (Fungi); Obelidium, *Obelidium mucronatum* KAI9342551 (Fungi); Gorgonomyces, *Gorgonomyces haynaldii* KAI8912823 (Fungi); Monosiga, *Monosiga brevicollis* XP_001743131 (Choanoflagellata); Sorex, *Sorex araneus* XP_004609762 (Animals). Amino acids that are identical and biochemically similar in all but one protein are labeled by a black and grey background, respectively.

What is the situation with TPPP-like proteins? My BLAST search shows that *A. protococcorum* does have, and *A. occidentale* does not have TPPP. Genomic and proteomic data for two different strains of *A. protococcorum,* X5, and FD95, were available. Both of them possess two long-type TPPPs (KAI3631655 and KAI3639621 in strain X5, KAI3650757 and KAI3652328 in strain FD95), which are almost identical to each other both within and between the strains (Table S1). A similar phenomenon occurred in the case of the green algae genus, *Ostreococcus*, which—unlike other green algae, such as *Chlamydomonas*—lost its flagellum but contained a highly divergent TPPP ortholog [4]. Long-type (i.e., animal-type) TPPPs are featured by the presence of a complete p25alpha domain (Figure 1). They occur in Opisthokonta (e.g., Choanoflagellata, animals, and some species of flagellated fungi) (Figure 2). The sequences of TPPPs in *A. protococcorum* are most similar to the RKP02545 protein of Chytridiomycota fungus, *Caulochytrium protostelioides* (Table S1). Interestingly, RKP02545 is a fungal-type TPPP, similar to the other three proteins of fungi among the 27 best hits listed in Table S1. All the other proteins are of animal origin.

**Figure 3:**
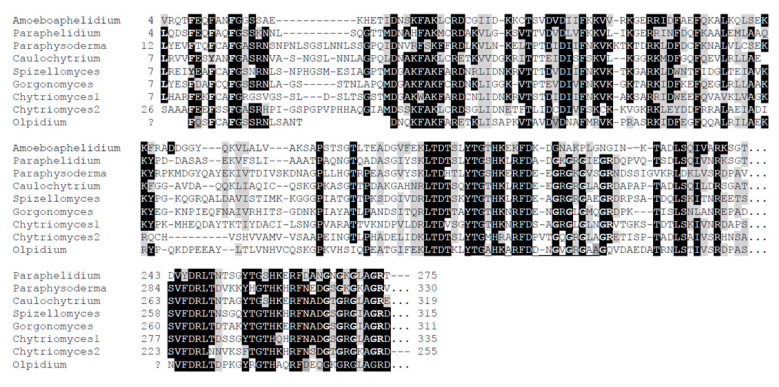
Comparison of the sequence of *Amoeboaphelidium protococcorum* TPPP with those of some fungal-type TPPPs. The multiple alignment (manually refined) of the sequences of p25alpha domains was conducted by Clustal Omega [15]. The N-termini (amino acids before the p25alpha domain) and the interdomain parts are not included in the alignment. Amoeboaphelidium, *A. protococcorum* strain X5 KAI3631655; Paraphelidium, *Paraphelidium tribonematis* TRINITY_DN24782; Paraphysoderma, *Paraphysoderma sedebokerense* KAI9140125; Caulochytrium, *Caulochytrium protostelioides* RKP02545; Spizellomyces, *Spizellomyces punctatus* XP_016604112; Gorgonomyces, *Gorgonomyces haynaldii* KAI8912588; Chytriomyces1, *Chytriomyces confervae* TPX65886; Chytriomyces2, *Chytriomyces confervae* TPX72533; Olpidium, *Olpidium bornovanus* KAG5460860 + KAG5458366. Amino acids that are identical and biochemically similar in at least three quarters of the fungal-type proteins are labeled by a black and grey background, respectively. The “Rossman-like” sequences, GXGXGXXGR, and the LXXF(Y)XXF(Y)XXF sequence at the beginning of the p25alpha domain are indicated by bold letters.

An NCBI Blast search was not possible for *P. tribonematis* as there were no *P. tribonematis* data on this site. However, its metatranscriptomic nucleotide contig assembly and the predicted proteome were deposited in figshare [13]. My analysis of the proteome revealed that *P. tribonematis* contained a fungal-type TPPP (TRINITY_DN24782_c0_g1_i2|m.37417), which showed a high homology with proteins of this type (Table S2, Figure 3). This type of TPPP is specific to flagellated fungi and was previously found in the phyla Chytridiomycota, Blastocladiomycota, and Olpidiomycota [6,9]. Within Chytridiomycota, most species of class Chytridiomycetes contain three kinds of TPPPs: an animal-type and two fungal-types [6].

**Figure 4:**
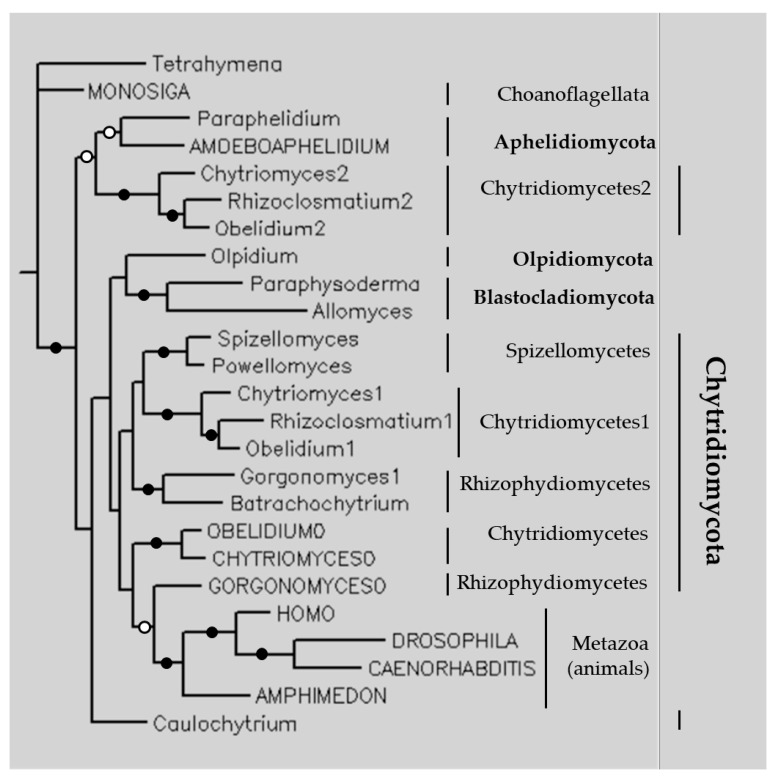
The phylogenetic tree of some TPPPs constructed by Bayesian analysis [16]. The number of generations was 1.4 × 10^−6^. Full and open circles at a node indicate that the branch was supported by the maximal Bayesian posterior probability (BPP) and ≥0.95 BPP, respectively. All the other branches were supported by BPP ≥ 0.5. The accession numbers of proteins are listed in Table S3. Uppercase letters indicate animal-type TPPPs, lowercase letters indicate fungal-type ones, except for the outgroup *Tetrahymena thermophila* TPPP (XP_001023601) (phylum Ciliophora), which is a short-type TPPP. Fungal phyla are indicated by bold letters.

The phylogenetic tree of TPPPs of fungi was constructed using the Bayesian method (Figure 4). In addition to the TPPPs of Fungi (fungal- and animal-types), some animal-type TPPPs of Metazoa and Choanoflagellata were also included. Proteins of the fungi/Metazoa group were separated from the *Monosiga brevicollis* (Choanoflagellata) protein (a long-type TPPP). The long (animal)-type TPPPs of the fungi/Metazoa group formed a distinct clade within which proteins of animal (*Amphimedon*, *Caenorhabditis*, *Drosophila*, *Homo*) and of fungal origin were separated from each other. This suggests that the fungal-type TPPP was not present in the common ancestor of Opisthokonta, as it is more parsimonious to imagine that it evolved in the common ancestor of fungi than to assume that this type of TPPP was independently lost in Choanoflagellata and Metazoa.

The only exception is the *Amoeboaphelidium* long-type TPPP that formed a clade with the fungal type in *Paraphelidium*. Phyla (Aphelidiomycota, Chytridiomycota, Blastocladiomycota, and Olpidiomycota) and the classes (Spizellomycetes, Chytridiomycetes, Rhizophydiomycetes) formed distinct clades according to their phylogeny. *Caulochytrium* has a separate position on our Bayesian tree. The exact position of this species has long been disputed; it was even claimed without any molecular evidence that ‘Caulochytriomycota’ formed a separate phylum [20]. New evidence strongly suggests that it is included in Chytridiomycota as a sister to class Chytridiomycetes [21] or Synchytriomycetes [22].

**Figure 5:**
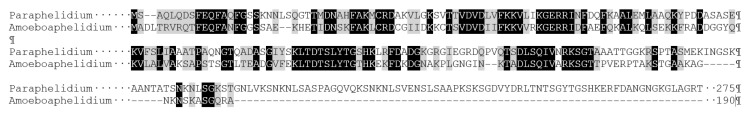
Sequence alignment of *Paraphelidium tribonematis* TRINITY_DN24782 and *Amoeboaphelidium protococcorum* KAI3631655 proteins by Clustal Omega [15]. Amino acids that are identical and biochemically similar are labeled by a black and grey background, respectively.

Interestingly, Aphelidiomycota TPPPs are sisters to a clade of Chytridiomycete fungal-type TPPPs. First, the two Aphelidiomycota TPPPs formed a common clade with each other, although one (*P. tribonematis*) contained two p25alpha domains and the other (*A. protococcorum*) only one. Based on the sequence alignment (Figure 5), they contained identical and biochemically similar amino acids in 47% and in 66%, respectively (cf. Table S2). It means that comparing these values with those of Table S1, *P. tribonematis* TPPP showed a higher homology with *A. protococcorum* TPPP than any other protein. (*P. tribonematis* TPPP is not involved in Table S1 since it is absent in the NCBI database.)

In addition, species in Chytridiomycetes contain two fungal-type paralogs labeled ‘1’ and ‘2’ in Figure 4. These paralogs are found in different clades, within which they are more similar to each other than to other TPPPs in the same species. The existence of the two groups has already been recognized [6]. They can be considered “outparalogs” [23] since the duplication events happened earlier than the species speciation, perhaps in the common ancestor of Chytridiomycetes.

These recent data on Aphelidiomycota strengthen the correlation between the incidence of p25alpha domain-containing proteins and that of the eukaryotic flagellum. (I do not discuss here or whether Aphelidiomycota or Aphelida is the correct name depending on whether there is a sister relationship [18] between Aphelida and ‘true’ Fungi, or Aphelidiomycota is considered a part of the fungal kingdom [10,22]. Moreover, the place of Aphelidiomycota/Aphelida is the same on the phylogenetic tree obtained by both groups, and the monophyly of Aphelida and Fungi is shown [24]. The presence of a fungal-type TPPP in *P. tribonematis* is an interesting addition to the phylogenetic classification of Aphelidiomycota.)

The other six phyla of flagellated fungi contain one or more TPPP-like proteins [6,19]. One exception occurs in the phylum Neocallimastigomycota: the flagellated anaerobic gut fungus, *Orpinomyces* sp. strain C1A, has no TPPP-like proteins. However, its closest relatives in this phylum have apicortin, a TPPP-like protein containing a partial p25alpha domain and a DCX domain (Pfam03607 or IPR003533). It should be noted that the genome of *Orpinomyces* sp. is 94% complete [25], and a TPPP-like gene can still be identified. On the other hand, TPPP-like proteins do not practically occur in terrestrial, non-flagellated fungi. A total of 1571 terrestrial (non-flagellated) fungi are available on the MycoCosm webpage (https://mycocosm.jgi.doe.gov/mycocosm/home) [26]. Only four possess a TPPP-like protein, namely, apicortin, which occurs in a family (Endogonaceae) of a relatively early branched terrestrial (i.e., non-flagellated) fungal phylum, Mucoromycota [6]. This may be a relic since no other p25alpha domain-containing protein was found in other terrestrial fungi.

*P. tribonematis* of Aphelidiomycota retains the flagellum and possesses a fungal-type TPPP containing both a complete and a partial p25alpha domain. If the flagellum has been lost for a long time (e.g., terrestrial fungi), these proteins cannot be found even in traces that contrast the flagellated species. Sometimes they were preserved as ‘relics’ in species at smaller phylogenetic distances (e.g., *A. protococcorum*); in which case they may acquire a new function. This suggestion is in accordance with the fact that the zoospore of *A. protococcorum* possesses a pseudocilium: a permanent immotile posterior projection containing microtubules, which may be considered a reduced posterior flagellum [27].

## Data Availability

The data presented in this study are available in this paper and in the Supplementary Material.

## Supplementary Materials

The following supporting information can be downloaded at: https://www.mdpi.com/article/10.3390/jof9030376/s1, Table S1. Best protein hits when using Amoeboaphelidium protococcorum KAI3631655.1 as a query. BLASTP search on NCBI protein database. Table S2. Best protein hits when using Paraphelidium tribonematis TPPP (TRINITY_DN24782_c0_g1_i2|m.37417) as a query. BLASTP search on NCBI protein database. Table S3. Accession numbers of proteins shown in Figure 4.
