# Supplementary material for: Tubulin Polymerization Promoting Proteins (TPPPs) of Aphelidiomycota: Correlation between the Incidence of p25alpha Domain and the Eukaryotic Flagellum
Source: J Fungi (Basel). 2023 Mar 19;9(3):376. doi: 10.3390/jof9030376 (PMC10057920; doi:10.3390/jof9030376)
Supplement: Supplementary file 1 [file jof-09-00376-s001.zip › jof-2239479-supplementary.pdf]

Supplementary Table S1. Best protein hits when using *Amoeboaphelidium protococcarum* KAI3631655.1 as a query. BLASTP search on NCBI protein database.

Protein	Species	Max Score	Total Score	Query Cover	E value	Per. Ident	Length	Accession
hypothetical protein MIR68_010128	*Amoeboaphelidium protococcarum*	354	354	100%	1e-122	100.00%	190	KAI3631655.1
hypothetical protein MP228_004238	*Amoeboaphelidium protococcarum*	346	346	100%	2e-119	96.84%	190	KAI3650757.1
hypothetical protein MP228_003631	*Amoeboaphelidium protococcarum*	343	343	100%	4e-118	96.32%	190	KAI3652328.1
hypothetical protein MIR68_002315	*Amoeboaphelidium protococcarum*	340	340	100%	6e-117	96.32%	190	KAI3639621.1
hypothetical protein CXG81DRAFT_17821	*Caulochytrium protostelioides*	130	130	85%	7e-33	43.43%	319	RKP02545.1
PREDICTED: tubulin polymerization-promoting protein family member 2	*Sorex araneus*	128	128	87%	2e-33	44.44%	171	XP_004609762.1
tubulin polymerization-promoting protein family member 2	*Suncus etruscus*	125	125	86%	2e-32	43.20%	171	XP_049624639.1
tubulin polymerization-promoting protein family member 2	*Jaculus jaculus*	124	124	82%	3e-32	45.34%	170	XP_004672419.1
p25-alpha-domain-containing protein	*Obelidium mucronatum*	124	124	81%	3e-31	42.42%	238	KAI9342285.1
p25-alpha-domain-containing protein	*Polychytrium aggrega*	121	121	84%	1e-30	42.70%	327	KAI9199861.1
TPPP2 protein	*Amazona guildingii*	120	120	73%	1e-30	46.53%	173	NXK77861.1
Tubulin polymerization-promoting protein family member 2	*Colius striatus*	120	120	83%	2e-30	44.17%	170	KFP31923.1
PREDICTED: tubulin polymerization-promoting protein family member 2-like	*Colius striatus*	120	120	83%	2e-30	44.17%	168	XP_010200717.1
tubulin polymerization-promoting protein family member 2	*Ochotona princeps*	119	119	82%	2e-30	43.48%	169	XP_004584952.1
tubulin polymerization-promoting protein family member 2	*Acomys russatus*	119	119	86%	3e-30	42.94%	170	XP_050998982.1
tubulin polymerization-promoting protein family member 2	*Mus pahari*	119	119	82%	3e-30	44.72%	170	XP_021059760.1
tubulin polymerization-promoting protein family member 2	*Phodopus roborovskii*	119	119	82%	3e-30	44.44%	170	XP_051032834.1
tubulin polymerization-promoting protein family member 2-like protein	*Amazona aestiva*	119	119	73%	5e-30	46.53%	168	KQK78373.1
p25-alpha-domain-containing protein	*Chytriomyces cf. hyalinus*	118	118	82%	3e-30	39.53%	237	KAI8842263.1
tubulin polymerization-promoting protein family member 2 isoform X2	*Phocoena sinus*	118	118	72%	7e-30	46.48%	171	XP_032478726.1
tubulin polymerization-promoting protein family member 2 isoform X3	*Delphinapterus leucas*	118	118	72%	7e-30	45.77%	165	XP_030618809.1
tubulin polymerization-promoting protein family member 2 isoform X1	*Myodes glareolus*	118	118	82%	8e-30	45.06%	170	XP_048291596.1
tubulin polymerization-promoting protein family member 2 isoform X3	*Zalophus californianus*	118	118	85%	8e-30	43.45%	171	XP_027426320.1
tubulin polymerization-promoting protein family member 2 isoform X4 [	*Delphinapterus leucas*	118	118	72%	8e-30	45.77%	164	XP_030618812.1
tubulin polymerization-promoting protein family member 2	*Neogale vison*	118	118	85%	9e-30	43.45%	171	XP_044086477.1
PREDICTED: tubulin polymerization-promoting protein family member 2	*Condylura cristata*	118	118	85%	1e-29	42.86%	171	XP_004694739.1
tubulin polymerization-promoting protein family member 2	*Eumetopias jubatus*	118	118	85%	1e-29	43.45%	171	XP_027950984.1
tubulin polymerization-promoting protein family member 2 isoform X1	*Phocoena sinus*	118	118	72%	1e-29	46.48%	180	XP_032478725.1
tubulin polymerization-promoting protein family member 2	*Marmota flaviventris*	118	118	72%	1e-29	46.85%	169	XP_027778204.1
tubulin polymerization-promoting protein family member 2 isoform X2	*Ailuropoda melanoleuca*	118	118	85%	1e-29	43.45%	171	XP_011233827.1
tubulin polymerization-promoting protein family member 2 isoform X2	*Delphinapterus leucas*	118	118	72%	1e-29	45.77%	180	XP_030618808.1

E-value is the measure of likeliness that sequence similarity is not by random chance. An E-value smaller than 1e-50 includes database matches of very high quality. Blast hits with E-value smaller than 0.01 can still be considered as good hit for homology matches. Yellow and green background indicate *Amoeboaphelidium* and other Fungi species, respectively. All other data applies to animal (vertebrate) species.

Supplementary Table S2.

Best protein hits when using *Paraphelidium tribonemae* TPPP (TRINITY_DN24782_c0_g1_i2|m.37417) as a query. BLASTP search on NCBI protein database.

Protein	Species	Max Score	Total Score	Query Cover	E value	Per. Ident	Length	Accession
p25-alpha-domain-containing protein	*Zopfochytrium polystomum*	201	250	98%	3e-60	41.96%	304	KAI9357369.1
p25-alpha-domain-containing protein	*Polychytrium aggregatum*	190	247	98%	8e-56	40.40%	327	XP_052963844.1
hypothetical protein HDV00_008104	*Rhizophlyctis rosea*	188	244	98%	4e-55	41.08%	319	KAJ3031848.1
hypothetical protein CXG81DRAFT_17821	*Caulochytrium protostelioides*	187	241	98%	6e-55	39.07%	319	RKP02545.1
p25-alpha-domain-containing protein	*Paraphysoderma sedebokerense*	183	183	98%	4e-53	39.80%	330	KAI9140125.1
hypothetical protein MIR68_002315	*Amoeboaphelidium protococcarum*	168	212	74%	4e-52	47.15%	190	KAI3639621.1
Tubulin polymerization-promoting protein member 3	*Clydaea vesicula*	178	178	98%	2e-51	38.98%	314	KAJ3223392.1
p25-alpha-domain-containing protein	*Gaertneriomyces semiglobifer*	176	231	98%	2e-50	39.73%	312	KAI9004733.1
hypothetical protein HDU86_003085	*Geranomyces michiganensis*	175	233	98%	2e-50	39.72%	314	KAJ3158133.1
hypothetical protein MP228_003631	*Amoeboaphelidium protococcarum*	164	208	74%	2e-50	45.83%	190	KAI3652328.1
hypothetical protein HDU96_003259	*Phlyctochytrium bullatum*	175	175	98%	3e-50	39.86%	318	KAJ3113561.1
hypothetical protein MP228_004238	*Amoeboaphelidium protococcarum*	164	207	74%	3e-50	45.83%	190	KAI3650757.1
hypothetical protein HDU85_000879	*Gaertneriomyces sp.*	174	229	98%	5e-50	38.85%	301	KAJ3185965.1
hypothetical protein HDU87_003629	*Geranomyces variabilis*	174	231	98%	5e-50	39.16%	314	KAJ3178317.1
hypothetical protein HK097_002729	*Rhizophlyctis rosea*	174	231	98%	7e-50	39.07%	321	KAJ3039857.1
uncharacterized protein SPPG_08463	*Spizellomyces punctatus*	174	228	98%	1e-49	39.38%	315	XP_016604112.1
hypothetical protein HDU84_006415	*Entophlyctis sp.*	173	229	98%	1e-49	38.52%	310	KAJ3379779.1
hypothetical protein HDU83_002002	*Entophlyctis luteolus*	173	228	98%	2e-49	38.52%	310	KAJ3356088.1
hypothetical protein HDU82_000297	*Entophlyctis luteolus*	173	228	98%	2e-49	38.52%	310	KAJ3199598.1
hypothetical protein HDU84_006465	*Entophlyctis sp.*	172	228	98%	5e-49	38.54%	310	KAJ3379674.1
hypothetical protein MIR68_010128	*Amoeboaphelidium protococcarum*	159	203	68%	1e-48	48.48%	190	KAI3631655.1
hypothetical protein HDU89_005536	*Geranomyces variabilis*	171	227	98%	2e-48	38.81%	314	KAJ3155973.1
hypothetical protein HDU83_004219	*Entophlyctis luteolus*	170	226	98%	2e-48	38.19%	310	KAJ3345323.1
p25-alpha-domain-containing protein	*Geranomyces variabilis*	171	227	98%	2e-48	38.81%	314	KAI8589706.1
hypothetical protein SpCBS45565_g07343	*Spizellomyces sp. 'palustris'*	171	219	98%	3e-48	37.70%	336	TPX61118.1
hypothetical protein HKX48_003590	*Thoreauomyces humboldtii*	167	225	98%	3e-47	39.66%	314	KAJ3017344.1
hypothetical protein HK100_002929	*Physocladia obscura*	167	221	98%	3e-47	36.33%	312	KAJ3142486.1
p25-alpha-domain-containing protein	*Powellomyces hirtus*	167	221	98%	3e-47	39.44%	303	KAI8908161.1
hypothetical protein PhCBS80983_g03683	*Powellomyces hirtus*	166	219	98%	8e-47	39.08%	303	TPX57673.1
p25-alpha-domain-containing protein	*Fimicolochytrium jonesii*	164	217	98%	3e-46	41.28%	304	XP_052926454.1
hypothetical protein HDU98_010151	*Podochytrium sp.*	164	217	98%	7e-46	35.57%	328	KAJ3066530.1
45 kDa subunit of RNA polymerase II	*Entophlyctis luteolus*	170	226	98%	2e-45	38.19%	647	KAJ3209884.1
p25-alpha-domain-containing protein	*Gorgonomyces haynaldii*	162	215	98%	3e-45	38.54%	311	KAI8912588.1
p25-alpha-domain-containing protein	Zopfochytrium polystomum	161	161	98%	5e-45	38.65%	287	KAI9325442.1
uncharacterized protein BATDEDRAFT_89639	Batrachochytrium dendrobatidis	160	160	95%	1e-44	38.03%	289	XP_006680205.1
hypothetical protein BDEG_26054	Batrachochytrium dendrobatidis	160	160	95%	1e-44	38.03%	299	OAJ42613.1

E-value is the measure of likeliness that sequence similarity is not by random chance. An E-value smaller than 1e-50 includes database matches of very high quality. Blast hits with E-value smaller than 0.01 can still be considered as good hit for homology matches. Yellow and green backgrounds indicate Aphelidiomycota and Blastocladiomycota species, respectively. All other data applies to Chytridiomycota species.

Table S3. Accession Numbers of fungal proteins shown in Figure 4.

Name on Figure 4	Species	Phylum	NCBI Accession No.
Allomyces	*Allomyces macrogynus*	Blastocladiomycota	KNE68590
AMOEBOAPHELIDIUM	*Amoeboaphelidium protococcarum*	Aphelidiomycota	KAI3631655
Batrachochytrium	*Batrachochytrium dendrobatidis*	Chytridiomycota	XP_006680205
CHYTRIOMYCES0	*Chytriomyces confervae*	Chytridiomycota	TPX78276
Chytriomyces1	*Chytriomyces confervae* 1	Chytridiomycota	TPX65886
Chytriomyces2	*Chytriomyces confervae* 2	Chytridiomycota	TPX72533
Caulochytrium	*Caulochytrium protostelioides*	Chytridiomycota	RKP02545
GORGONOMYCES0	*Gorgonomyces haynaldii*	Chytridiomycota	KAI8912823
Gorgonomyces1	*Gorgonomyces haynaldii*	Chytridiomycota	KAI8912588
OBELDIUM0	*Obelidium mucronatum*	Chytridiomycota	KAI9342551
Obelidium1	*Obelidium mucronatum*	Chytridiomycota	KAI9351240
Obelidium2	*Obelidium mucronatum*	Chytridiomycota	KAI9342285
Olpidium	*Olpidium bornovanus*	Olpidiomycota	KAG5460860+ KAG5458366
Paraphelidium	*Paraphelidium tribonemae*	Aphelidiomycota	TRINITY_DN24782[1]
Paraphysoderma	*Paraphysoderma sedebokerense*	Blastocladiomycota	KAI9140125
Powellomyces	*Powellomyces hirtus*	Chytridiomycota	TPX57673
Rhizoclosmatium1	*Rhizoclosmatium globosum*	Chytridiomycota	KAJ3297182
Rhizoclosmatium2	*Rhizoclosmatium globosum*	Chytridiomycota	ORY45507
Spizellomyces	*Spizellomyces punctatus*	Chytridiomycota	XP_016604112
MONOSIGA	*Monosiga brevicollis*	Choanoflagellata	XP_001743131
AMPHIMEDON	*Amphimedon queenslandica*	Porifera (sponges)	XP_003384590
CAENORHABDITIS	*Caenorhabditis elegans*	Nematode	NP_491219
DROSOPHILA	*Drosophila melanogaster*	Arthropoda	NP_648881
HOMO	*Homo sapiens*	Chordata	NP_776245
Tetrahymena	*Tetrahymena*	Ciliophora	XP_001023601

[1]Taken from https://figshare.com/articles/dataset/Commun_Biol_aphelid_datasets/7339469/1
